# A hierarchical Naïve Bayes Model for handling sample heterogeneity in classification problems: an application to tissue microarrays

**DOI:** 10.1186/1471-2105-7-514

**Published:** 2006-11-24

**Authors:** Francesca Demichelis, Paolo Magni, Paolo Piergiorgi, Mark A Rubin, Riccardo Bellazzi

**Affiliations:** 1 Bionformatics, SRA, ITC-irst & Dept. of Information and Communication Technology, University of Trento, Trento, Italy; 2 Department of Pathology, Brigham and Women's Hospital, Boston, MA, USA; 3 Harvard Medical School, Boston, MA, USA; 4 Dipartimento di Informatica e Sistemistica, Università di Pavia, Pavia, Italy; 5 Dana Farber Harvard Cancer Center, Boston, MA, USA

## Abstract

**Background:**

Uncertainty often affects molecular biology experiments and data for different reasons. Heterogeneity of gene or protein expression within the same tumor tissue is an example of biological uncertainty which should be taken into account when molecular markers are used in decision making. Tissue Microarray (TMA) experiments allow for large scale profiling of tissue biopsies, investigating protein patterns characterizing specific disease states. TMA studies deal with multiple sampling of the same patient, and therefore with multiple measurements of same protein target, to account for possible biological heterogeneity. The aim of this paper is to provide and validate a classification model taking into consideration the uncertainty associated with measuring replicate samples.

**Results:**

We propose an extension of the well-known Naïve Bayes classifier, which accounts for biological heterogeneity in a probabilistic framework, relying on Bayesian hierarchical models. The model, which can be efficiently learned from the training dataset, exploits a closed-form of classification equation, thus providing no additional computational cost with respect to the standard Naïve Bayes classifier. We validated the approach on several simulated datasets comparing its performances with the Naïve Bayes classifier. Moreover, we demonstrated that explicitly dealing with heterogeneity can improve classification accuracy on a TMA prostate cancer dataset.

**Conclusion:**

The proposed Hierarchical Naïve Bayes classifier can be conveniently applied in problems where within sample heterogeneity must be taken into account, such as TMA experiments and biological contexts where several measurements (replicates) are available for the same biological sample. The performance of the new approach is better than the standard Naïve Bayes model, in particular when the within sample heterogeneity is different in the different classes.

## Background

The biomedical sciences are fraught with uncertainty. The sources of this uncertainty are manifold. Devices used to monitor biological processes vary in terms of resolutions. Gaps in the full understanding of basic biology compound this problem. Biological diversity or heterogeneity may make predictions difficult. Finally, uncertainty may be due to the unpredictable sources of noise, which can be inside or outside the biological system itself.

In molecular biology uncertainty is ubiquitous; for example, tissue heterogeneity makes it difficult to compare a tissue sample composed of pure tumor cell populations with one composed of tumor and other non-tumoral elements such as supporting structural tissues (i.e. stroma) and vessels. However, in molecular biology, one rarely can examine an entire tumor and biopsies are taken with the assumption that they represent a portion of the whole tumor.

This paper addresses the uncertainty associated with measuring replicate samples. Understanding such kind of uncertainty would help guide decision making and allow for alternate strategies to be explored. Usually, the measurements of replicate samples are averaged to derive a single measurement. This value is then used for example when building a classification system which may play a critical role in the decision making process. Unfortunately, an average measurement (or median value) hides the uncertainty or heterogeneity present in the replicates, and may thus lead to decision making rules which are too reliant on this pooled data. This process may lead to a model that is not sufficiently robust to work in an independent dataset.

TMA studies represent a context where the issue of biological heterogeneity is particularly relevant. Where gene expression microarray experiments provide researchers with quantitative evaluation of transcripts, TMA evaluate DNA, RNA or protein targets through *in situ *investigations (analyses performed on tissues). *In situ *evaluations are characterized by the fact that the morphology of the analyzed samples is intact and therefore the potential biological heterogeneity within tumor tissue can be analyzed. TMAs allow for large scale profiling of tissue samples. For example, TMAs can be used to investigate panels of proteins that may play a role in tumor progression. They have the potential to be easily translatable to a clinical application such as the development of diagnostic biomarkers, e.g., AMACR [1] or to access a therapeutic target, e.g., Her-2-neu [2].

TMA datasets usually include replicate core biopsies of the same tissue from the same individual to ensure that enough representative tissue is available in each experiment and to better represent the biological variability of the tissue itself and of the protein activity (i.e. accurate sampling). Most TMA datasets are evaluated using straightforward pooling of the data from replicates, thus ignoring variations among biopsies from the same patient (the so called within sample variability). The mean, the maximum or the minimum is usually adopted and the strategy may be based on biological knowledge or on known protein associations. However, it has been found that different choices can lead to covariates with different significance levels in Cox regression [3]. Interestingly, when multiple biomarkers are evaluated, one approach is chosen and applied for all of them regardless of the biologic implications.

However the degree of heterogeneity of the tumor tissue may be an important biological parameter. In a probabilistic framework, for example, accounting for the within sample variability caused by the tumor tissue heterogeneity, could alter the probability of a case belonging to a certain class (even changing the predicted class), providing insight into the particular case study. When measurement occurs at different levels, i.e. different biopsies of the same tumor or different tumors, standard statistical techniques are not appropriate because they either assume that groups belong to entirely different populations or ignore the aggregate information entirely.

Hierarchical models (multilevel models) provide a way of pooling the information for the different groups without assuming that they belong to precisely the same population [4]. They are typically used when information is available but the observation units differ (i.e., meta-analysis of separate randomized trials).

Herein we propose a classification model, which accounts for the tumor within sample variability in a probabilistic framework, relying on Bayesian hierarchical models. Hierarchical Bayes models have been used for modeling individual effects in several experimental contexts, ranging from toxicology to tumor risk [4]. For this reason, their use in the classification context seems particularly suitable to handle TMA data and tumor heterogeneity.

The paper is structured as follows: we first provide relevant background on Bayesian classifiers (specifically on the Naïve Bayes classifiers) and on Bayesian hierarchical models. Then we describe the proposed method and compare its performances to a Naive Bayesian classifier, in which we applied standard pooling strategies. The results will be shown on simulated datasets characterized by different ratios of within and between samples variability and on a real classification problem based on TMA data we generated in our laboratory from a prostate cancer progression array (TMA Core of Dana Farber Harvard Cancer Center, Boston, MA) developed to identify proteins that can distinguish aggressive from indolent forms of this common tumor type.

### Bayesian classifiers and the Naïve Bayes

In this paper we focus on classification problems, where, given the data coming from a target case, we must decide to which class the case belongs. For example, given the set of tumor marker values measured on biopsies of a tissue of a given patient, we must decide if the patient is affected by a particular kind of tumor.

From a Bayesian viewpoint, a classification problem can be written as the problem of finding the class with maximum probability given a set of observed attribute values. Such probability is seen as the posterior probability of the class given the data, and is usually computed using the Bayes theorem, as *P*(*C*|***X***) = *P*(***X***|*C*) *P*(*C*)/*P*(***X***), where *C *is any of the possible class values, ***X ***is a vector of *N*_*feature *_attribute values, while *P*(*C*) and *P*(***X***|*C*) are the prior probability of the class and the conditional probability of the attribute values given the class, respectively. Usually Bayesian classifiers maximize *P*(***X***|*C*)*P*(*C*), which is proportional to *P*(*C*|***X***), being *P*(***X***) constant given a dataset.

Bayesian classifiers are known to be the optimal classifiers, since they minimize the risk of misclassification. However, they require defining *P*(***X***|*C*), i.e. the *joint *probability of the attributes given the class. Estimating this probability distribution from a training dataset is a difficult problem, because it may require a very large dataset even for a moderate number of attributes in order to significantly explore all the possible combinations.

Conversely, in the framework of Naïve Bayes classifiers, the attributes are assumed to be independent from each other given the class. This allows us to write, following Bayes theorem, the posterior probability of the class *C *as: *p*(*C*|***X***) = ∏_*l *= 1_^*Nfeature *^*p*(**X**^*l*^|*C*) *p*(*C*)/*P*(***X***). The Naïve Bayes classifier is therefore fully defined simply by the conditional probabilities of each attribute given the class. The conditional independence property largely simplifies the learning process of the model from data. In presence of discrete and Gaussian data this process turns out to be straightforward. Despite its simplicity, the Naïve Bayes classifier is known to be a robust method, which shows on average good performance in terms of classification accuracy, also when the independence assumption does not hold [5,6]. Due to its fast induction, the Naïve Bayes classifier is often considered as a reference method in classification studies. Several approaches have been proposed to generalize such classifier [7] and there has been a recent interest in applying hierarchical models to Bayesian classification, such as in the field of expression array analysis [8,9]. In this paper we present a step forward, describing a hierarchical Naïve Bayesian model which can be convenientln used for classification purposes in the presence of replicated measurements, as for TMA data.

### Bayesian Hierarchical Models

Bayesian hierarchical models (BHM) [4] are powerful instruments able to describe complex interactions between the parameters of a stochastic model. In particular, BHMs are often used to describe population models, in which the parameters characterizing the model of an individual are considered to be related to the parameters of the other individuals belonging to the same population. In this paper we will cope with the problem of classifying tumors of different patients for which repeated measurements of tumor markers are available. The probability distribution of such measurements will depend on the patient; however, all the patients suffering from the same disease will be assumed to be related to each other in terms of tumor marker probability distributions.

Bayesian hierarchical models provide a natural way to represent this relationship by specifying a suitable conditional independence structure and a suitable set of conditional probability distributions.

The simpler structure of a BHM can be summarized as follows: let us suppose that a certain variable *x *(e.g. a tumor marker) has been measured *n*_*rep *_times in *m *patients belonging to the same population, i.e. they have the same tumor type. Let us also suppose that *x *is a stochastic variable depending on a set of parameter *θ*, so that for the *i*-th subject, such dependency is expressed by the probability *p*(*x*_i_| *θ*_*i*_). The assumption that the individuals are "related" to each other can be then represented by introducing the conditional probability *p*(*θ*_*i*_|*ϕ*), where *ϕ *is a set of hyper-parameters typical of a population. In this way, each subject is characterized by a probability distribution that depends on population parameters which are common for all individuals of the same population. If we assign a prior distribution to *ϕ*, say *p*(*ϕ*), the joint prior distribution will be *p*(*ϕ*,*θ*) = *p*(*θ *|*ϕ*)*p*(*ϕ*), where *θ *= {*θ*_1_, *θ*_2_, ..., *θ*_*m*_}. Once a data set ***X ***= {***X***_1_,..., ***X***_*m*_} is observed on all *m *patients, where **X**_*i *_= (*x*_*i*1_, *x*_*i*2_,..., xinrepi
 MathType@MTEF@5@5@+=feaafiart1ev1aaatCvAUfKttLearuWrP9MDH5MBPbIqV92AaeXatLxBI9gBaebbnrfifHhDYfgasaacH8akY=wiFfYdH8Gipec8Eeeu0xXdbba9frFj0=OqFfea0dXdd9vqai=hGuQ8kuc9pgc9s8qqaq=dirpe0xb9q8qiLsFr0=vr0=vr0dc8meaabaqaciaacaGaaeqabaqabeGadaaakeaacqWG4baEdaWgaaWcbaGaemyAaKMaemOBa42aaSbaaWqaaiabdkhaYjabdwgaLjabdchaWjabdMgaPbqabaaaleqaaaaa@36CD@) is the measurement vector for the *i*-th patient, the joint posterior distribution *p*(*ϕ*, *θ*|***X***) can be computed by applying the Bayes theorem. It is easy to show that such distribution is proportional to *p*(***X***|*θ*) *p*(*θ*|*ϕ*) *p*(*ϕ*). Since the population parameters are usually unknown, the integral of such equation over *ϕ *allows to calculate the posterior distributions for the model parameters *θ *given the data coming from all patients.

BHMs have been applied in a variety of contexts, ranging from signal processing [10] to medicine and pharmacology [11,12] and to bioinformatics. [13-16]. Moreover, several computational techniques for specifying and fitting BHMs have been introduced also to deal with discrete responses, multivariate models, survival models and time series models. Useful reviews can be found in papers and books [17,18]. Recently, hierarchical models have been applied to extend the Naïve Bayes model in order to relax the assumptions of conditional independence between attributes [19]. In our paper we will use hierarchical models to handle repeated measurements and their heterogeneity.

## Results

### The Hierarchical Naïve Bayes approach

From a probabilistic perspective, a classification problem can be viewed as the selection of the class which has the highest (posterior) probability given the available data. Here we explicitly handle data with multiple replicate values.

In the case of TMA, we can think of one case as being the tumor tissue of one patient and replicates being the multiple biopsies from that patient. Therefore, the evaluation of a target protein (feature) on a TMA section will provide the pathologist with multiple evaluations of protein expression for each patient (case).

Let xijlk
 MathType@MTEF@5@5@+=feaafiart1ev1aaatCvAUfKttLearuWrP9MDH5MBPbIqV92AaeXatLxBI9gBaebbnrfifHhDYfgasaacH8akY=wiFfYdH8Gipec8Eeeu0xXdbba9frFj0=OqFfea0dXdd9vqai=hGuQ8kuc9pgc9s8qqaq=dirpe0xb9q8qiLsFr0=vr0=vr0dc8meaabaqaciaacaGaaeqabaqabeGadaaakeaacqWG4baEdaqhaaWcbaGaemyAaKMaemOAaOgabaGaemiBaWMaem4AaSgaaaaa@33CA@ be the *j*-th replicate measurement of the *l*-th feature of the *i*-th case corresponding to class *k*. For the sake of simplicity, given a class *k *and a feature *l*, we will write it as *x*_*ij*_, *j *= 1,...,*n*_*rep*_, *i *= 1,...,*N*_*Ck*_, where *N*_*Ck *_is the number of cases in the class *C*_*k*_.

**Figure 1:**
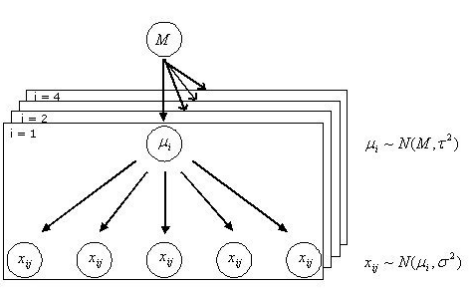
**Structure of a hierarchical model**. The replicates *j *of the generic subject *i *are normally distributed around a mean value *μ*_*i *_with a within sample variance *σ*^2^, i.e. *x*_*ij*_~*N*(*μ*_*i*_, *σ*^2^). The mean values *μ*_*i *_are normally distributed around a "population" mean value *M *with between sample variance *τ*^2^, i.e. *μ*_*i*_~*N*(*M*, *τ*^2^).

Let us assume that the values of replicates of the generic case *i *are normally distributed around a mean value *μ*_*i *_with a variance *σ*^2 ^(independent from *i*, but dependent on the class *k*), i.e. *x*_*ij*_~*N*(*μ*_*i*_, *σ*^2^). The mean values *μ*_*i *_are, at their turn, normally distributed around a "population" mean value *M *with variance *τ*^2^, i.e. *μ*_*i*_~*N*(*M*, *τ*^2^). The assumption that the variance is the same for all patients belonging to the same class reflects the intuitive notion that the variability over replicates, due for example to the tissue heterogeneity, is a property of the disease. Such an assumption, which is realistic in TMA data, turns out to be convenient when estimating the variance from the data: the reliability of the estimate is increased by the higher number of measurements exploited. The resulting hierarchical model is presented in Fig. 1.

Given this probabilistic model, we here describe how to classify a new case, supposing that the class model parameters *M*, *τ*^2^, *σ*^2 ^are known for each class. In the Methods section we detail how to learn the model parameters from a training dataset. Moreover, the same section reports the classification and the learning phase of Standard Naïve Bayes (StNB) classifier, in order to highlight the differences.

To classify a new case in the Bayesian framework it is necessary to evaluate the posterior probability of each class given the case data. Let us define the vector **X**_*i *_= (*x*_*i1*_, *x*_*i*2_,..., *x*_*inrepi*_), which represents the replicate measurements of the *i*-th case for a given feature (univariate case). For sake of simplicity, we omit the sub-index *i *hereafter.

By applying the Bayes' theorem, the posterior probability for the class C_k _given the set of data is

P(Ck|X,σ2,M,τ2)=P(X|Ck,σ2,M,τ2)P(Ck)p(X)∝P(X|Ck,σ2,M,τ2)P(Ck).
 MathType@MTEF@5@5@+=feaafiart1ev1aaatCvAUfKttLearuWrP9MDH5MBPbIqV92AaeXatLxBI9gBaebbnrfifHhDYfgasaacH8akY=wiFfYdH8Gipec8Eeeu0xXdbba9frFj0=OqFfea0dXdd9vqai=hGuQ8kuc9pgc9s8qqaq=dirpe0xb9q8qiLsFr0=vr0=vr0dc8meaabaqaciaacaGaaeqabaqabeGadaaakeaacqWGqbaucqGGOaakcqWGdbWqdaWgaaWcbaGaem4AaSgabeaakiabcYha8Hqabiab=HfayjabcYcaSGGaciab+n8aZnaaCaaaleqabaGaeGOmaidaaOGaeiilaWIaemyta0KaeiilaWIae4hXdq3aaWbaaSqabeaacqaIYaGmaaGccqGGPaqkcqGH9aqpdaWcaaqaaiabdcfaqjabcIcaOiab=HfayjabcYha8jabdoeadnaaBaaaleaacqWGRbWAaeqaaOGaeiilaWIae43Wdm3aaWbaaSqabeaacqaIYaGmaaGccqGGSaalcqWGnbqtcqGGSaalcqGFepaDdaahaaWcbeqaaiabikdaYaaakiabcMcaPiabdcfaqjabcIcaOiabdoeadnaaBaaaleaacqWGRbWAaeqaaOGaeiykaKcabaGaemiCaaNaeiikaGIae8hwaGLaeiykaKcaaiabg2Hi1kabdcfaqjabcIcaOiab=HfayjabcYha8jabdoeadnaaBaaaleaacqWGRbWAaeqaaOGaeiilaWIae43Wdm3aaWbaaSqabeaacqaIYaGmaaGccqGGSaalcqWGnbqtcqGGSaalcqGFepaDdaahaaWcbeqaaiabikdaYaaakiabcMcaPiabdcfaqjabcIcaOiabdoeadnaaBaaaleaacqWGRbWAaeqaaOGaeiykaKIaeiOla4caaa@74D9@

To evaluate the posterior probability, the marginal likelihood *P*(***X***| *C*_*k*_, *σ*^2^, *M*, *τ*^2^) can be computed exploiting the conditional independence assumptions described in the hierarchical model of Figure 1 as:

P(X|Ck,σ2,M,τ2)=∫μP(X|μ,σ2)P(μ|M,τ2)dμ.
 MathType@MTEF@5@5@+=feaafiart1ev1aaatCvAUfKttLearuWrP9MDH5MBPbIqV92AaeXatLxBI9gBaebbnrfifHhDYfgasaacH8akY=wiFfYdH8Gipec8Eeeu0xXdbba9frFj0=OqFfea0dXdd9vqai=hGuQ8kuc9pgc9s8qqaq=dirpe0xb9q8qiLsFr0=vr0=vr0dc8meaabaqaciaacaGaaeqabaqabeGadaaakeaacqWGqbaucqGGOaakieqacqWFybawcqGG8baFcqWGdbWqdaWgaaWcbaGaem4AaSgabeaakiabcYcaSGGaciab+n8aZnaaCaaaleqabaGaeGOmaidaaOGaeiilaWIaemyta0KaeiilaWIae4hXdq3aaWbaaSqabeaacqaIYaGmaaGccqGGPaqkcqGH9aqpdaWdrbqaaiabdcfaqjabcIcaOiab=HfayjabcYha8jab+X7aTjabcYcaSiab+n8aZnaaCaaaleqabaGaeGOmaidaaOGaeiykaKIaemiuaaLaeiikaGIae4hVd0MaeiiFaWNaemyta0KaeiilaWIae4hXdq3aaWbaaSqabeaacqaIYaGmaaGccqGGPaqkcqWGKbazcqGF8oqBcqGGUaGlaSqaaiab+X7aTbqab0Gaey4kIipaaaa@5D68@

The marginal likelihood can be written as (for sake of readability the subscript *k *and the model parameters *M*, *τ*^2^, *σ*^2 ^have been omitted in the left hand side of the equation):

P(X|C)=1(σ2π)nrep(τ2π)*∫μexp⁡(−12σ2∑j(xj−μ)2−12τ2(μ−M)2)dμ.
 MathType@MTEF@5@5@+=feaafiart1ev1aaatCvAUfKttLearuWrP9MDH5MBPbIqV92AaeXatLxBI9gBaebbnrfifHhDYfgasaacH8akY=wiFfYdH8Gipec8Eeeu0xXdbba9frFj0=OqFfea0dXdd9vqai=hGuQ8kuc9pgc9s8qqaq=dirpe0xb9q8qiLsFr0=vr0=vr0dc8meaabaqaciaacaGaaeqabaqabeGadaaakeaacqWGqbaucqGGOaakieqacqWFybawcqGG8baFcqWGdbWqcqGGPaqkcqGH9aqpdaWcaaqaaiabigdaXaqaamaabmaabaacciGae43Wdm3aaOaaaeaacqaIYaGmcqGFapaCaSqabaaakiaawIcacaGLPaaadaahaaWcbeqaaiabd6gaUnaaBaaameaacqWGYbGCcqWGLbqzcqWGWbaCaeqaaaaakmaabmaabaGae4hXdq3aaOaaaeaacqaIYaGmcqGFapaCaSqabaaakiaawIcacaGLPaaaaaGaeiOkaOYaa8quaeaacyGGLbqzcqGG4baEcqGGWbaCaSqaaiab+X7aTbqab0Gaey4kIipakmaabmaabaGaeyOeI0YaaSaaaeaacqaIXaqmaeaacqaIYaGmcqGFdpWCdaahaaWcbeqaaiabikdaYaaaaaGcdaaeqbqaaiabcIcaOiabdIha4naaBaaaleaacqWGQbGAaeqaaOGaeyOeI0Iae4hVd0MaeiykaKYaaWbaaSqabeaacqaIYaGmaaaabaGaemOAaOgabeqdcqGHris5aOGaeyOeI0YaaSaaaeaacqaIXaqmaeaacqaIYaGmcqGFepaDdaahaaWcbeqaaiabikdaYaaaaaGccqGGOaakcqGF8oqBcqGHsislcqWGnbqtcqGGPaqkdaahaaWcbeqaaiabikdaYaaaaOGaayjkaiaawMcaaiabdsgaKjab+X7aTjabc6caUaaa@7425@

Applying simple algebra (details are reported Additional file 1), we obtain:

P(X|C)=σ(2πσ)nrepnrepτ2+σ2exp⁡−(∑jxj22σ2+M22τ2)*exp⁡((τ2nrep2xmean2σ2+σ2M2τ2+2nrepxmeanM)2(nrepτ2+σ2)).
 MathType@MTEF@5@5@+=feaafiart1ev1aaatCvAUfKttLearuWrP9MDH5MBPbIqV92AaeXatLxBI9gBaebbnrfifHhDYfgasaacH8akY=wiFfYdH8Gipec8Eeeu0xXdbba9frFj0=OqFfea0dXdd9vqai=hGuQ8kuc9pgc9s8qqaq=dirpe0xb9q8qiLsFr0=vr0=vr0dc8meaabaqaciaacaGaaeqabaqabeGadaaakeaacqWGqbaucqGGOaakieqacqWFybawcqGG8baFcqWGdbWqcqGGPaqkcqGH9aqpdaWcaaqaaGGaciab+n8aZbqaamaabmaabaWaaOaaaeaacqaIYaGmcqGFapaCaSqabaGccqGFdpWCaiaawIcacaGLPaaadaahaaWcbeqaaiabd6gaUnaaBaaameaacqWGYbGCcqWGLbqzcqWGWbaCaeqaaaaakmaakaaabaGaemOBa42aaSbaaSqaaiabdkhaYjabdwgaLjabdchaWbqabaGccqGFepaDdaahaaWcbeqaaiabikdaYaaakiabgUcaRiab+n8aZnaaCaaaleqabaGaeGOmaidaaaqabaaaaOGagiyzauMaeiiEaGNaeiiCaaNaeyOeI0YaaeWaaeaadaWcaaqaamaaqafabaGaemiEaG3aa0baaSqaaiabdQgaQbqaaiabikdaYaaaaeaacqWGQbGAaeqaniabggHiLdaakeaacqaIYaGmcqGFdpWCdaahaaWcbeqaaiabikdaYaaaaaGccqGHRaWkdaWcaaqaaiabd2eannaaCaaaleqabaGaeGOmaidaaaGcbaGaeGOmaiJae4hXdq3aaWbaaSqabeaacqaIYaGmaaaaaaGccaGLOaGaayzkaaGaeiOkaOIagiyzauMaeiiEaGNaeiiCaa3aaeWaaeaadaWcaaqaaiabcIcaOmaalaaabaGae4hXdq3aaWbaaSqabeaacqaIYaGmaaGccqWGUbGBdaqhaaWcbaGaemOCaiNaemyzauMaemiCaahabaGaeGOmaidaaOGaemiEaG3aa0baaSqaaiabd2gaTjabdwgaLjabdggaHjabd6gaUbqaaiabikdaYaaaaOqaaiab+n8aZnaaCaaaleqabaGaeGOmaidaaaaakiabgUcaRmaalaaabaGae43Wdm3aaWbaaSqabeaacqaIYaGmaaGccqWGnbqtdaahaaWcbeqaaiabikdaYaaaaOqaaiab+r8a0naaCaaaleqabaGaeGOmaidaaaaakiabgUcaRiabikdaYiabd6gaUnaaBaaaleaacqWGYbGCcqWGLbqzcqWGWbaCaeqaaOGaemiEaG3aaSbaaSqaaiabd2gaTjabdwgaLjabdggaHjabd6gaUbqabaGccqWGnbqtcqGGPaqkaeaacqaIYaGmcqGGOaakcqWGUbGBdaWgaaWcbaGaemOCaiNaemyzauMaemiCaahabeaakiab+r8a0naaCaaaleqabaGaeGOmaidaaOGaey4kaSIae43Wdm3aaWbaaSqabeaacqaIYaGmaaGccqGGPaqkaaaacaGLOaGaayzkaaGaeiOla4caaa@ADAC@

Finally, given the model parameters, the new case ***X ***can be classified into the class that maximize the posterior probability, which is proportional to the marginal likelihood if the classes are *a priori *equally likely.

It is interesting to note that the main novelty of the method is that the classification rule (through the marginal likelihood) includes the information on the within sample heterogeneity. Such information is expressed by the parameter *σ*^2^, which may therefore guide decisions when there is a clear difference between the within sample heterogeneity of cases belonging to the different classes. Let us note that standard approaches, such as the StNB or the quadratic discriminant analysis, can take into account only between samples variability, expressed in our model by the parameter *τ*^2 ^. Moreover, since the classification rule can be calculated in closed-form, it can be used in real-time applications, such as the StNB classifier.

The generalization for the multivariate case, i.e. X¯
 MathType@MTEF@5@5@+=feaafiart1ev1aaatCvAUfKttLearuWrP9MDH5MBPbIqV92AaeXatLxBI9gBaebbnrfifHhDYfgasaacH8akY=wiFfYdH8Gipec8Eeeu0xXdbba9frFj0=OqFfea0dXdd9vqai=hGuQ8kuc9pgc9s8qqaq=dirpe0xb9q8qiLsFr0=vr0=vr0dc8meaabaqaciaacaGaaeqabaqabeGadaaakeaaieqacuWFybawgaqeaaaa@2E03@ = (**X**^1^, **X**^2^**,...,X**^*Nfeature*^), can be obtained by assuming, as in the StNB classifier, the conditional independence of the features given the class, i.e. P(X¯|C)=∏l=1NfeatureP(Xl|C)
 MathType@MTEF@5@5@+=feaafiart1ev1aaatCvAUfKttLearuWrP9MDH5MBPbIqV92AaeXatLxBI9gBaebbnrfifHhDYfgasaacH8akY=wiFfYdH8Gipec8Eeeu0xXdbba9frFj0=OqFfea0dXdd9vqai=hGuQ8kuc9pgc9s8qqaq=dirpe0xb9q8qiLsFr0=vr0=vr0dc8meaabaqaciaacaGaaeqabaqabeGadaaakeaacqWGqbaucqGGOaakieqacuWFybawgaqeaiabcYha8jabdoeadjabcMcaPiabg2da9maarahabaGaemiuaaLaeiikaGIae8hwaG1aaWbaaSqabeaacqWGSbaBaaGccqGG8baFcqWGdbWqcqGGPaqkaSqaaiabdYgaSjabg2da9iabigdaXaqaaiabd6eaojabdAgaMjabdwgaLjabdggaHjabdsha0jabdwha1jabdkhaYjabdwgaLbqdcqGHpis1aaaa@4CEE@

In this case, the posterior probability of class *k *is

P(Ck|X¯)=P(X¯|Ck)P(Ck)P(X¯)∝∏l=1NfeatureP(Xl|Ck)P(Ck).
 MathType@MTEF@5@5@+=feaafiart1ev1aaatCvAUfKttLearuWrP9MDH5MBPbIqV92AaeXatLxBI9gBaebbnrfifHhDYfgasaacH8akY=wiFfYdH8Gipec8Eeeu0xXdbba9frFj0=OqFfea0dXdd9vqai=hGuQ8kuc9pgc9s8qqaq=dirpe0xb9q8qiLsFr0=vr0=vr0dc8meaabaqaciaacaGaaeqabaqabeGadaaakeaacqWGqbaucqGGOaakcqWGdbWqdaWgaaWcbaGaem4AaSgabeaakiabcYha8Hqabiqb=HfayzaaraGaeiykaKIaeyypa0ZaaSaaaeaacqWGqbaucqGGOaakcuWFybawgaqeaiabcYha8jabdoeadnaaBaaaleaacqWGRbWAaeqaaOGaeiykaKIaemiuaaLaeiikaGIaem4qam0aaSbaaSqaaiabdUgaRbqabaGccqGGPaqkaeaacqWGqbaucqGGOaakcuWFybawgaqeaiabcMcaPaaacqGHDisTdaqeWbqaaiabdcfaqjabcIcaOiab=HfaynaaCaaaleqabaGaemiBaWgaaOGaeiiFaWNaem4qam0aaSbaaSqaaiabdUgaRbqabaGccqGGPaqkcqWGqbaucqGGOaakcqWGdbWqdaWgaaWcbaGaem4AaSgabeaakiabcMcaPaWcbaGaemiBaWMaeyypa0JaeGymaedabaGaemOta4KaemOzayMaemyzauMaemyyaeMaemiDaqNaemyDauNaemOCaiNaemyzauganiabg+GivdGccqGGUaGlaaa@6A08@

### Results on simulated and real data

We present the results we obtained using both computationally generated datasets and a real TMA protein expression dataset.

A first set of simulated data was generated to represent the best scenario, by incrementally varying the within sample variance *σ*^2 ^for one class only. A second set of normally distributed data was generated using a variety of parameter values (see Additional file 2). The training and classification properties of the proposed algorithm were evaluated in both cases. Finally, we analyzed a set of real TMA protein expression data, in order to evaluate the potentials of the method for the analysis of real data.

In both studies, being the Hierarchical Naïve Bayes (HierNB) classifier an extension of the StNB classifier to cope with replicate measurements, results are compared with the StNB classifier to highlight, without introducing additional bias due to the different classification techniques, the advantage of the new approach. However, the real data were analyzed by several other classification methods and the results are reported in the Additional file 3.

The classifiers are compared on the basis of two indexes: the accuracy (defined as the ratio of the properly classified and the total classified cases) and the Brier score [20], measuring the difference between the probability of an event and its occurrence, expressed as 0 or 1 depending on if the event has occurred or not. Confidence intervals of accuracy are evaluated by repeating several times learning and classification after a suitable data randomization [21]. Moreover, in the case of the real TMA dataset, we use sensitivity (defined as the ratio of the true positive classified cases and all positive cases), specificity (defined as the ratio of the true negative classified cases and all negative cases) and the area under the ROC curve [21].

The data analysis reported in this paper has been implemented in the R statistical package [22].

### Simulated data

#### Data description

We generated simulated datasets with 4000 patients (2000 for class 1 and 2000 for class 2), 5 replicates each and a number of independent features ranging from 1 to 10. For each feature, the values of the five replicates were randomly extracted from a normal distribution with fixed variance *σ*^2 ^(dependent on the class and on the feature) and mean randomly generated from a normal distribution with fixed mean M and variance *τ*^2^, again dependent on the class and on the feature.

A first set of experiments was run for the univariate case (one feature), so that the two classes basically had the same parameter values, but for the within sample variance parameter *σ*^2 ^that is assumed bigger in the second class. In particular they had a similar population mean M and exactly the same class variance *τ*^2 ^(M_1 _= 100, M_2 _= 105, *τ*_1_^2 ^= *τ*_2_^2 ^= 300, *σ*_1_^2 ^= 10). The parameter *σ*_2_^2 ^varied from 15 to 75.

A second set of experiments was run simulating both univariate case studies and multivariate case studies (here we report results for two, three and ten features). The values of the first feature were generated for all these experiments using 90, 300 and 100 as M, *τ*^2 ^and *σ*^2 ^for class 1 and 140, 600 and 400 as M, *τ*^2 ^and *σ*^2 ^for class 2.

The complete set of parameter values used for the model in the multivariate set of experiment is reported in the Additional file 2.

We assessed the performances of the two classifiers by equally dividing the dataset into training and test set.

#### Data results

The results of the first experiment are shown in Tables 1. There is an enormous advantage (both in term of accuracy and Brier score) in the HierNB model, respect to the standard approach, where basically no distinction can be made between the two classes (the two classes are not well separated). The advantage of the HierNB increases as the difference between the variance of the replicates in the two classes increases. This first experiment shows that the proposed method is able to extend the current classification approaches by taking advantage from the information which can be derived from within sample heterogeneity.

**Table 1:** Results on simulated data for 1 feature with different level of within sample heterogeneity in the different classes.

		HierNB Classifier		StNB Classifier	
Exp	*σ*_2_^2^	Acc	Brier	Acc	Brier

1	15	0.620 [0.604 0.636]	0.449	0.559 [0.539 0.580]	0.490
2	30	0.762 [0.740 0.790]	0.307	0.560 [0.540 0.580]	0.490
3	45	0.830 [0.813 0.849]	0.228	0.554 [0.537 0.570]	0.490
4	60	0.878 [0.866 0.888]	0.176	0.556 [0.531 0.582]	0.490
5	75	0.899 [0.883 0.914]	0.147	0.560 [0.534 0.586]	0.490

Exp = experiment number, Acc = Accuracy, Brier = Brier Score. In brackets the 95% confidence intervals for the estimate of the accuracy.

Results for the second set of four experiments are presented in Table 2. For each experiment we report the number of features, the accuracy and Brier Score for both the HierNB and the StNB classifier.

**Table 2:** Results on simulated data: experiments were done using increasing number of features.

		HierNB Classifier		StNB Classifier	
Exp	N Feat.	Acc	Brier	Acc	Brier

1	1	0.925 [0.917, 0.933]	0.112	0.874 [0.864, 0.884]	0.184
2	2	0.966 [0.960, 0.971]	0.052	0.921 [0.912, 0.929]	0.118
3	3	0.987 [0.983, 0.990]	0.020	0.946 [0.938, 0.952]	0.082
4	10	0.998 [0.997, 0.999]	0.002	0.985 [0.981, 0.989]	0.023

Exp = number of experiment, N. Feat = Number of features, Acc = Accuracy, Brier = Brier Score. In brackets the 95% confidence intervals for the estimate of the accuracy.

The HierNB classifier performs better in all the experiments, showing higher accuracy and lower Brier score.

**Figure 2:**
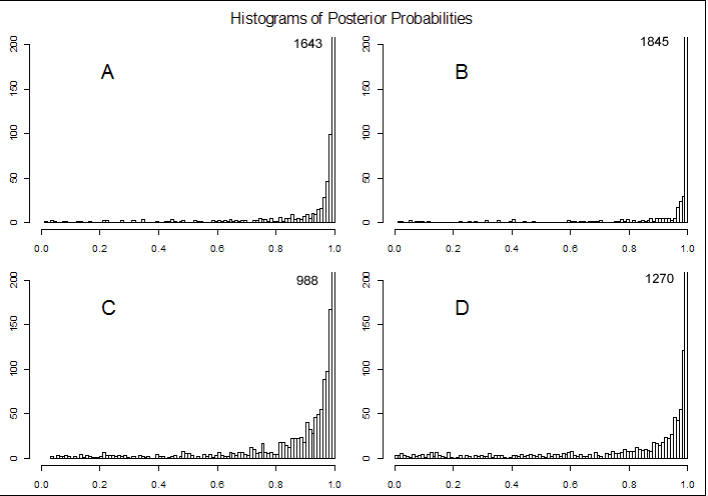
**Posterior probabilities of the three feature simulated experiment**. Histograms of posterior probabilities of the three feature experiment (Exp.3, Table 2) on a simulated dataset. Panels A and B show the results obtained with the HierNB classifier for class 1 and 2 respectively; panels C and D show results obtained with the StNB classifier. In the upper right corner of each panel the frequency of the bin corresponding to the highest posterior probability range is reported.

In Figure 2, the posterior probabilities of both the classifiers evaluated on the test datasets of one experiment (Table 2, experiment #3) are shown.

The HierNB classifier shows a better separation between the two classes not only in term of accuracy but also in term of credibility of the classification (as highlighted by the Brier Score). The confidence intervals of the estimated accuracy confirm that in all the experiments the proposed method outperforms the standard classifier. This second experiment shows that, even if the experimental context is complex since the classes show an overlap due to the values of the within and between sample variability, the method is able to perform equal or better than the StNB.

### Real data

#### Data description

We used a research dataset obtained from a recently constructed prostate progression TMA, as previously described [23].

The TMA was constructed to test molecular differences between localized and metastatic prostate cancer samples, on a total of 288 core biopsies. In this paper, we explore the expression of two proteins, i.e. EZH2 and AMACR known to be differentially expressed in non aggressive or localized tumors (class 1 or negative class) versus aggressive or metastatic prostate cancers (class 2 or positive class). The Polycomb Group protein, EZH2, is over-expressed in hormone-refractory and metastatic prostate cancer [24] and may play a role in the progression of prostate cancer as well as serve as a marker distinguishing indolent prostate cancer from those at risk of lethal progression. *α*-Methylacyl CoA racemase (AMACR) is a biomarker that was identified by both differential display and expression array analysis as a gene abundantly expressed in prostate cancer relative to benign prostate epithelium [25-27]. AMACR is used as a clinical marker to diagnose prostate cancer [1]. Prostate cancers that produce lower levels of AMACR have a worse clinical outcome even after controlling for other clinical parameters [28].

The TMA dataset includes 72 patients (samples), 36 for each class, each case having four replicates. After the processing of the TMA slides, 35 and 34 cases were suitable for analysis for class 1 and class 2 respectively (69 cases) and each case was characterized measuring from 1 to 4 times for two proteins. The assumption that the data have a Gaussian distribution given the class has been verified by applying the Kolmogorov-Smirnov test. Table 3 describes the dataset through the model parameters *M *and *τ*^2 ^estimated according to the StNB and *σ*^2 ^estimated as the average within case variance for the two classes. Since the estimate of *σ*^2 ^is different in the two classes, this classification problem may benefit from the use of the HierNB approach.

**Table 3:** TMA data description: model parameters of localized (class 1) and metastatic prostate cancer tumors (class 2) for two proteins.

	M		*τ*^2^		*σ*^2^	
Class	1	2	1	2	1	2

AMACR	155.2	148.8	201.2	208.8	49.1	85.7
EZH2Int	146.2	141.6	86.7	107.9	135.7	53.9

M = class mean; *τ*^2 ^= class variance; *σ*^2 ^= averaged within sample variance.

We assessed the performances of the two classifiers by applying one hundred times a 10 fold cross-validation procedure with different fold randomization and then by computing the average results.

#### Data results

**Figure 3:**
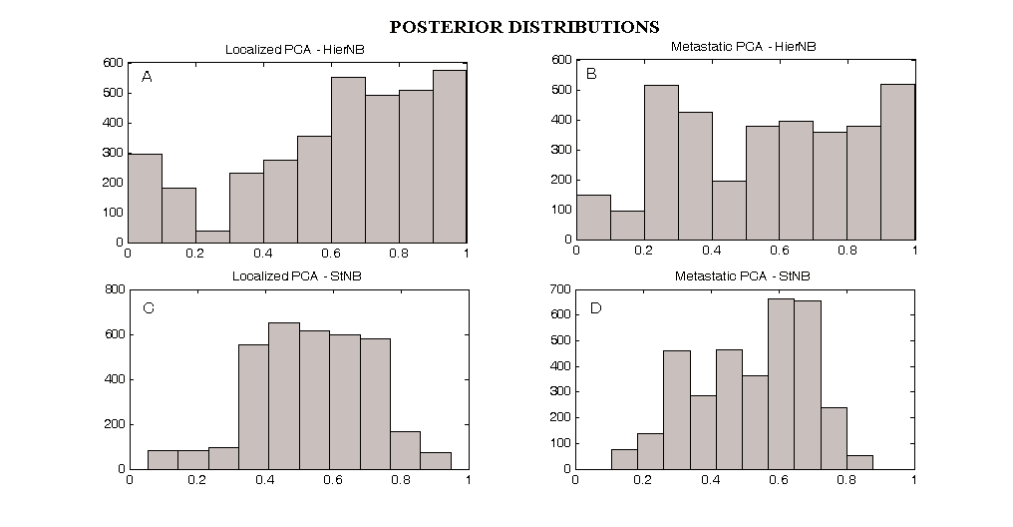
**Posterior probabilities of prostate cancer cases**. Histograms of the posterior probabilities of prostate cancer cases. Panels A and B show the results evaluated with the HierNB classifier for class 1 (localized tumors) and 2 (aggressive tumors) respectively; panels C and D show results evaluated with the StNB classifier.

Results obtained for the prostate cancer dataset are presented in Table 4. We report accuracy, specificity, sensitivity, area under the ROC curve (ROC curves are shown in the Additional file 4) and Brier Score for the HierNB and for the StNB classifier. In Figure 3, we report histograms of posterior probabilities obtained by the two models.

**Table 4:** Results on TMA dataset for the two proteins.

	**Acc**	**Spec**	**Sens**	**AUC**	**Brier**
**HierNB Model**	0.65 [0.62–0.68]	0.71 [0.66–0.74]	0.60 [0.56–0.62]	0.69 [0.689–0.693]	0.41 [0.39–0.42]
**StNB Model**	0.58 [0.54–0.61]	0.58 [0.54–0.62]	0.57 [0.53–0.61]	0.62 [0.617–0.622]	0.47 [0.46–0.48]

Acc = Accuracy, Spec = specificity, Sens = Sensitivity, Brier = Brier Score. In brackets the 95% confidence intervals for the estimates, AUC = area under the ROC.

The classification performance of the HierNB model clearly outperforms the StNB one, for what concerns all the evaluation parameters considered. In particular, both accuracy and the Brier score are significantly better in the HierNB case than in the StNB one, also considering the 95% confidence interval of the estimates. The fact that classification accuracy in distinguishing localized prostate cancer from metastases is only about 60% is not surprising. The complexity of this classification problem has been recently discussed in Bismar, Demichelis et al. [23].

The HierNB classifier also shows a significantly higher specificity, and a similar sensitivity. We note from the histogram of posterior probabilities (Figure 3) that the HierNB method has better performances for metastatic cancer (panel B) than the StNB approach (which shows uncertain classification for many patients, panel D).

## Discussion

Few studies have dealt with the problem of uncertain data in classification [29,30]. In the bioinformatics arena, several recent studies addressed the topic of uncertain data, in particular on DNA microarrays data. However, their main emphasis is related to the management of uncertainty when applying feature selection strategies [16,31]. Another example of handling uncertainty in classification is provided by Bhattacharyya et al., where they characterize each data point with an uncertainty model based on ellipsoids [32].

In this paper we have proposed a classifier based on Bayesian hierarchical models and have applied it on TMA datasets. The approach permits embedding in the classification model the tumor variability (heterogeneity of protein levels across tumor tissue), using the tuple of protein level measurements of each case instead of unique representative value as done by conventional approaches.

Bayesian hierarchical models have two main advantages with respect to other methods: i) they coherently manage uncertainty in the framework of probability theory; ii) they make explicit the assumptions which the model relies on. The implementation of the Bayesian classifier presented in this paper is an extension of the well known Naïve Bayes classifier. It assumes that all the attributes are independent among each other given the class. Moreover, we have also assumed that the probability distributions are conditionally Gaussian.

Preliminary performance tests on simulated data give us some clues about the applicability of the proposed model. With respect to classification, we observed that when classes have similar within sample variances no differences in terms of classification accuracy are obtained, as expected. However, increasing differences in the posterior distributions are detected as the difference of the within sample variability increases, e.g. *σ*_*1*_^*2*^<<*σ*_*2*_^2^. In this case the HierNB model outperforms the standard approach.

On TMA real data, we saw that the hierarchical model may improve specificity, which is part of the clinical question, and emphasizes the information available at every level, accounting for the spread of the replicate measures and thus may provide interesting insights into the biology of the tumor samples being analyzed. Rather interestingly, in this case the classification model is able to improve the data comprehension, highlighting if the heterogeneity of the tumor tissue sample is critical or not in the decision making process. Moreover, hierarchical models are also able to exploit the information on the lack of heterogeneity.

Heterogeneous and homogeneous protein expression may reflect different biological processes occurring in tumors. Exploiting this data may be critical in understanding the underlying biology.

Finally, the HierNB presents interesting robustness properties when comparing the results obtained in the data-rich case of the simulation study (4000 samples) with the relatively data-poor real one (69 samples). The real case is much more difficult than the simulated one, due to the lower number of samples and the smaller difference of the mean values of the markers in the two classes. Such a difficulty results in more spread posterior distributions and in lower accuracy and higher Brier score values of all tested classification models. However, in both the simulated and the real cases the HierNB shows nearly the same gain in accuracy with respect to the StNB, taking advantage from the within sample variability information to better separate classes.

From a practical point of view, in TMA experiments in which hundreds of cases are evaluated and only a fraction do not fit well into one class or another, one can imagine that by using the hierarchical Naïve Bayes model, cases with a posterior probability within a certain window around 0.5 would be classified as ambiguous and would require re-review.

From a methodological point of view, in order to generalize the proposed approach, we are now working on the following aspects:

1) Learning: while the classification step fully follows the Bayesian approach, the learning phase of the proposed method is not fully Bayesian. This choice was motivated by the need to perform fast learning from potentially large datasets for the needed probability distributions. However, it is also possible to resort to a more rigorous learning procedure by paying the price of implementing iterative procedures, such as Expectation Maximization (EM) or Monte Carlo Markov chain (MCMC) approaches [33]. Future versions of our tool will include also such kind of estimation algorithms [34].

2) Non Gaussian distributions: we have also implemented a version of the hierarchical Naïve Bayes approach for discrete variables, relying on multinomial and Dirichlet probability distributions [34,35]. This extension allows managing arbitrary data distributions after proper discretization.

## Conclusion

We have proposed a novel approach for dealing with uncertain data in classification, with applications to TMA microarrays. The proposed model has, as its unique property, the capability of handling data heterogeneity in a sound probabilistic way, without requiring additional computational burden with respect to the standard Naïve Bayes approach. Based on the results obtained on simulated and real data, we can conclude that the proposed approach is particularly useful when the within sample heterogeneity differs between classes. Its application to TMA data has been shown to provide more insight into the information available in the database and to improve the decision making process also in presence of a very limited number of features. The proposed model can be conveniently applied and extended to deal with other application domains in Bioinformatics.

## Methods

### Tissue microarray technology

TMAs were recently developed to facilitate tissue-based research [36]. TMAs can be used for any type of study where standard tissue slides had previously been used. However, they present numerous advantages [37]. TMAs allow for screening of a large number of tissue samples under similar experimental conditions (large scale process) while conserving tissue and resources. Typically a TMA block contains up to 600 tissue biopsies, depending on the needle diameter used to transfer the samples (from 0.6 to 2 mm). TMA sections are serially obtained at 4–5 micrometers thickness with a microtome. TMA sections are then processed as conventional histological tissue sections.

Cylindrical tissue biopsies are transferred with a biopsy needle from carefully selected morphologically representative areas from original paraffin blocks (donor blocks), each containing tumor tissue from a patient. Core tissue biopsies are then arrayed into a new "recipient" paraffin block by using a tissue arrayer using a precise spacing pattern along x and y axis, which generates a regular matrix of cores. Typically, more than one biopsy from each patient is included in a TMA block; replicates allow for good representation of the patient's tumor and to potentially detect heterogeneous expression of markers (e.g. proteins) of interest within the tumor. How well TMA samples represent entire tumors has been the focus of several recent studies [38]. The results of those studies are dependant on tumor types and study purposes. A biomarker with homogenous expression throughout the entire tumor will not require as many replicates as a biomarker that is only focally expressed by the target tissue.

### Learning the Hierarchical Naïve Bayes Model

The classification algorithm described in the Results section assumes that the model parameters (*M*, *τ*^*2*^, *σ*^*2*^) have been estimated from a training dataset. In the implementation of the method presented in this paper, we have adopted an approximation of the maximum likelihood estimation approach, called empirical learning [39].

Following such approach, the within sample means and variances are estimated as:

μ^i=∑jnrepxijnrepi
 MathType@MTEF@5@5@+=feaafiart1ev1aaatCvAUfKttLearuWrP9MDH5MBPbIqV92AaeXatLxBI9gBaebbnrfifHhDYfgasaacH8akY=wiFfYdH8Gipec8Eeeu0xXdbba9frFj0=OqFfea0dXdd9vqai=hGuQ8kuc9pgc9s8qqaq=dirpe0xb9q8qiLsFr0=vr0=vr0dc8meaabaqaciaacaGaaeqabaqabeGadaaakeaaiiGacuWF8oqBgaqcamaaBaaaleaacqWGPbqAaeqaaOGaeyypa0ZaaSaaaeaadaaeWbqaaiabdIha4naaBaaaleaacqWGPbqAcqWGQbGAaeqaaaqaaiabdQgaQbqaaiabd6gaUnaaBaaameaacqWGYbGCcqWGLbqzcqWGWbaCaeqaaaqdcqGHris5aaGcbaGaemOBa42aaSbaaSqaaiabdkhaYjabdwgaLjabdchaWnaaBaaameaacqWGPbqAaeqaaaWcbeaaaaaaaa@4623@ and σ^2=∑i=1NCk∑jnrep(xij−μ^i)2NCknrepi
 MathType@MTEF@5@5@+=feaafiart1ev1aaatCvAUfKttLearuWrP9MDH5MBPbIqV92AaeXatLxBI9gBaebbnrfifHhDYfgasaacH8akY=wiFfYdH8Gipec8Eeeu0xXdbba9frFj0=OqFfea0dXdd9vqai=hGuQ8kuc9pgc9s8qqaq=dirpe0xb9q8qiLsFr0=vr0=vr0dc8meaabaqaciaacaGaaeqabaqabeGadaaakeaaiiGacuWFdpWCgaqcamaaCaaaleqabaGaeGOmaidaaOGaeyypa0ZaaabCaeaadaWcaaqaamaaqahabaGaeiikaGIaemiEaG3aaSbaaSqaaiabdMgaPjabdQgaQbqabaGccqGHsislcuWF8oqBgaqcamaaBaaaleaacqWGPbqAaeqaaOGaeiykaKYaaWbaaSqabeaacqaIYaGmaaaabaGaemOAaOgabaGaemOBa42aaSbaaWqaaiabdkhaYjabdwgaLjabdchaWbqabaaaniabggHiLdaakeaacqWGobGtdaWgaaWcbaGaem4qam0aaSbaaWqaaiabdUgaRbqabaaaleqaaOGaemOBa42aaSbaaSqaaiabdkhaYjabdwgaLjabdchaWnaaBaaameaacqWGPbqAaeqaaaWcbeaaaaaabaGaemyAaKMaeyypa0JaeGymaedabaGaemOta40aaSbaaWqaaiabdoeadnaaBaaabaGaem4AaSgabeaaaeqaaaqdcqGHris5aaaa@5A4C@, while the population mean and variance as:

M^pool=∑iNCkμ^iσ^i2∑i1σ^i2
 MathType@MTEF@5@5@+=feaafiart1ev1aaatCvAUfKttLearuWrP9MDH5MBPbIqV92AaeXatLxBI9gBaebbnrfifHhDYfgasaacH8akY=wiFfYdH8Gipec8Eeeu0xXdbba9frFj0=OqFfea0dXdd9vqai=hGuQ8kuc9pgc9s8qqaq=dirpe0xb9q8qiLsFr0=vr0=vr0dc8meaabaqaciaacaGaaeqabaqabeGadaaakeaacuWGnbqtgaqcamaaBaaaleaacqWGWbaCcqWGVbWBcqWGVbWBcqWGSbaBaeqaaOGaeyypa0ZaaSaaaeaadaaeWbqaamaalaaabaacciGaf8hVd0MbaKaadaWgaaWcbaGaemyAaKgabeaaaOqaaiqb=n8aZzaajaWaa0baaSqaaiabdMgaPbqaaiabikdaYaaaaaaabaGaemyAaKgabaGaemOta40aaSbaaWqaaiabdoeadnaaBaaabaGaem4AaSgabeaaaeqaaaqdcqGHris5aaGcbaWaaabuaeaadaWcaaqaaiabigdaXaqaaiqb=n8aZzaajaWaa0baaSqaaiabdMgaPbqaaiabikdaYaaaaaaabaGaemyAaKgabeqdcqGHris5aaaaaaa@4CB1@ and τ^corr2=∑iNCk(μ^i−M^pool)2NCk−∑iNCk∑jnrep(xij−μ^i)2NCknrepi2
 MathType@MTEF@5@5@+=feaafiart1ev1aaatCvAUfKttLearuWrP9MDH5MBPbIqV92AaeXatLxBI9gBaebbnrfifHhDYfgasaacH8akY=wiFfYdH8Gipec8Eeeu0xXdbba9frFj0=OqFfea0dXdd9vqai=hGuQ8kuc9pgc9s8qqaq=dirpe0xb9q8qiLsFr0=vr0=vr0dc8meaabaqaciaacaGaaeqabaqabeGadaaakeaaiiGacuWFepaDgaqcamaaDaaaleaacqWGJbWycqWGVbWBcqWGYbGCcqWGYbGCaeaacqaIYaGmaaGccqGH9aqpdaWcaaqaamaaqahabaGaeiikaGIaf8hVd0MbaKaadaWgaaWcbaGaemyAaKgabeaakiabgkHiTiqbd2eanzaajaWaaSbaaSqaaiabdchaWjabd+gaVjabd+gaVjabdYgaSbqabaGccqGGPaqkdaahaaWcbeqaaiabikdaYaaaaeaacqWGPbqAaeaacqWGobGtdaWgaaadbaGaem4qam0aaSbaaeaacqWGRbWAaeqaaaqabaaaniabggHiLdaakeaacqWGobGtdaWgaaWcbaGaem4qam0aaSbaaWqaaiabdUgaRbqabaaaleqaaaaakiabgkHiTmaalaaabaWaaabCaeaadaaeWbqaaiabcIcaOiabdIha4naaBaaaleaacqWGPbqAcqWGQbGAaeqaaOGaeyOeI0Iaf8hVd0MbaKaadaWgaaWcbaGaemyAaKgabeaakiabcMcaPmaaCaaaleqabaGaeGOmaidaaaqaaiabdQgaQbqaaiabd6gaUnaaBaaameaacqWGYbGCcqWGLbqzcqWGWbaCaeqaaaqdcqGHris5aaWcbaGaemyAaKgabaGaemOta40aaSbaaWqaaiabdoeadnaaBaaabaGaem4AaSgabeaaaeqaaaqdcqGHris5aaGcbaGaemOta40aaSbaaSqaaiabdoeadnaaBaaameaacqWGRbWAaeqaaaWcbeaakiabd6gaUnaaDaaaleaacqWGYbGCcqWGLbqzcqWGWbaCdaWgaaadbaGaemyAaKgabeaaaSqaaiabikdaYaaaaaaaaa@7972@ where σ^i2=σ^2nrepi
 MathType@MTEF@5@5@+=feaafiart1ev1aaatCvAUfKttLearuWrP9MDH5MBPbIqV92AaeXatLxBI9gBaebbnrfifHhDYfgasaacH8akY=wiFfYdH8Gipec8Eeeu0xXdbba9frFj0=OqFfea0dXdd9vqai=hGuQ8kuc9pgc9s8qqaq=dirpe0xb9q8qiLsFr0=vr0=vr0dc8meaabaqaciaacaGaaeqabaqabeGadaaakeaaiiGacuWFdpWCgaqcamaaDaaaleaacqWGPbqAaeaacqaIYaGmaaGccqGH9aqpdaWcaaqaaiqb=n8aZzaajaWaaWbaaSqabeaacqaIYaGmaaaakeaacqWGUbGBdaWgaaWcbaGaemOCaiNaemyzauMaemiCaa3aaSbaaWqaaiabdMgaPbqabaaaleqaaaaaaaa@3C64@.

The estimate of the population variance includes two terms, representing *between *samples and *within *sample variances (expressing the inter-subject variability and the intra-subject variability, respectively). The estimate of *τ *is particularly critical: it is valid as far as the within sample variability is less than the between sample one, i.e. under the assumption that the measurements heterogeneity is not too large [4]. In case such assumption does not hold, other learning strategies can be conveniently applied, such as the EM estimate. We note that empirical learning allows the exploitation of the HierNB approach without additional computational burden with respect to the standard (non hierarchical) approach.

### Classification by standard Naïve Bayes Model

Also in this case the classification of a new case requires the evaluation of the posterior probability of each class given the case data. However, the standard Naïve Bayes classifier does not consider the replicate measurements, but only their aggregate value (standard pooling strategy, e.g. mean value). Let X be the mean value of a given feature (univariate case) of the case to classify. The likelihood of X, X ∈ R, is P(X|C)=12πτexp⁡(−12τ2(X−M)2)
 MathType@MTEF@5@5@+=feaafiart1ev1aaatCvAUfKttLearuWrP9MDH5MBPbIqV92AaeXatLxBI9gBaebbnrfifHhDYfgasaacH8akY=wiFfYdH8Gipec8Eeeu0xXdbba9frFj0=OqFfea0dXdd9vqai=hGuQ8kuc9pgc9s8qqaq=dirpe0xb9q8qiLsFr0=vr0=vr0dc8meaabaqaciaacaGaaeqabaqabeGadaaakeaacqWGqbaucqGGOaakcqWGybawcqGG8baFcqWGdbWqcqGGPaqkcqGH9aqpdaWcaaqaaiabigdaXaqaamaakaaabaGaeGOmaidcciGae8hWdahaleqaaOGae8hXdqhaaiGbcwgaLjabcIha4jabcchaWjabcIcaOiabgkHiTmaalaaabaGaeGymaedabaGaeGOmaiJae8hXdq3aaWbaaSqabeaacqaIYaGmaaaaaOGaeiikaGIaemiwaGLaeyOeI0Iaemyta0KaeiykaKYaaWbaaSqabeaacqaIYaGmaaGccqGGPaqkaaa@4BC2@, being *τ *and M, the variance and the mean of the distribution of the feature values in the class *C*. By Bayes' theorem, the posterior probability of each class can be easily evaluated and the new instance classified into the class with maximal posterior probability. The generalization for the multivariate case can be obtained by exploiting the assumption of conditional independence of the features given the class as discussed in the Background section.

### Learning of standard Naïve Bayes Model

Also in this case, the model parameters (M, *τ*^2^) have to be estimated from a training dataset. They can be computed as:

M^=∑iNCkμ^iNCk
 MathType@MTEF@5@5@+=feaafiart1ev1aaatCvAUfKttLearuWrP9MDH5MBPbIqV92AaeXatLxBI9gBaebbnrfifHhDYfgasaacH8akY=wiFfYdH8Gipec8Eeeu0xXdbba9frFj0=OqFfea0dXdd9vqai=hGuQ8kuc9pgc9s8qqaq=dirpe0xb9q8qiLsFr0=vr0=vr0dc8meaabaqaciaacaGaaeqabaqabeGadaaakeaacuWGnbqtgaqcaiabg2da9maalaaabaWaaabCaeaaiiGacuWF8oqBgaqcamaaBaaaleaacqWGPbqAaeqaaaqaaiabdMgaPbqaaiabd6eaonaaBaaameaacqWGdbWqdaWgaaqaaiabdUgaRbqabaaabeaaa0GaeyyeIuoaaOqaaiabd6eaonaaBaaaleaacqWGdbWqdaWgaaadbaGaem4AaSgabeaaaSqabaaaaaaa@3DBD@ and τ^2=∑iNCk(μ^i−M^)2NCk
 MathType@MTEF@5@5@+=feaafiart1ev1aaatCvAUfKttLearuWrP9MDH5MBPbIqV92AaeXatLxBI9gBaebbnrfifHhDYfgasaacH8akY=wiFfYdH8Gipec8Eeeu0xXdbba9frFj0=OqFfea0dXdd9vqai=hGuQ8kuc9pgc9s8qqaq=dirpe0xb9q8qiLsFr0=vr0=vr0dc8meaabaqaciaacaGaaeqabaqabeGadaaakeaaiiGacuWFepaDgaqcamaaCaaaleqabaGaeGOmaidaaOGaeyypa0ZaaSaaaeaadaaeWbqaaiabcIcaOiqb=X7aTzaajaWaaSbaaSqaaiabdMgaPbqabaGccqGHsislcuWGnbqtgaqcaiabcMcaPmaaCaaaleqabaGaeGOmaidaaaqaaiabdMgaPbqaaiabd6eaonaaBaaameaacqWGdbWqdaWgaaqaaiabdUgaRbqabaaabeaaa0GaeyyeIuoaaOqaaiabd6eaonaaBaaaleaacqWGdbWqdaWgaaadbaGaem4AaSgabeaaaSqabaaaaaaa@447E@.

## Authors' contributions

FD, PM and RB developed and implemented the Hierarchical Naïve Bayes Model presented in the paper. MAR was involved in the discussions about the suitability of the model to face protein expression heterogeneity in human tumors and generated the TMA protein expression datasets. PP helped in the evaluation of the model performances. All authors read and approved the final manuscript.

## Supplementary Material

Additional file 1 The marginal likelihood for the Hierarchical Naïve Bayes Model. Click here for file

Additional file 2 Parameter values used to generate simulated data. Click here for file

Additional file 3 Comparison of the proposed approach with other classification strategies on the TMA protein expression dataset. Click here for file

Additional file 4 ROC Curves for the TMA protein expression dataset as calculated by running 100 times 10-fold cross validation (Table 4 in the paper). Click here for file
