# Supplementary material for: A hierarchical Naïve Bayes Model for handling sample heterogeneity in classification problems: an application to tissue microarrays
Source: BMC Bioinformatics. 2006 Nov 24;7:514. doi: 10.1186/1471-2105-7-514 (PMC1698579; doi:10.1186/1471-2105-7-514)
Supplement: Additional file 1 — The marginal likelihood for the Hierarchical Naïve Bayes Model. [file 1471-2105-7-514-S1.doc]

**Additional file 1**

**The marginal likelihood for the Hierarchical Naïve Bayes Model**

The marginal likelihood *P(X | C_k_, σ^2^, M, τ^2^)* computed exploiting the conditional independence assumption of the Hierarchical Naïve Bayes Model can be written as:

$$P(\mathbf{X}|C)=\frac{1}{\left(\sigma\sqrt{2\pi}\right)^{n_{rep}}\left(\tau\sqrt{2\pi}\right)}*\int_{\mu} \exp\left(-\frac{1}{2\sigma^{2}}\sum_{j} (x_{j}-\mu)^{2}-\frac{1}{2\tau^{2}}(\mu-M)^{2}\right)d\mu.$$

For simplicity, we can write:

$P(\mathbf{X}|C)=\frac{1}{\left(\sqrt{2\pi}/S\right)^{n_{rep}}\left(\sqrt{2\pi}/T\right)}\int_{\mu} \exp\left(-\frac{S^{2}}{2}(\sum_{j} x_{j}^{2}+n_{rep}\mu^{2}-2\mu\sum_{j} x_{j})-\frac{T^{2}}{2}(\mu^{2}+M^{2}-2\mu M)\right)d\mu$,

where $S^{2}=\frac{1}{\sigma^{2}}$ and $T^{2}=\frac{1}{\tau^{2}}$.

$$P(\mathbf{X}|C)=\frac{\exp(-\frac{1}{2}(S^{2}\sum_{j} x_{j}^{2}+T^{2}M^{2}))}{\left(\sqrt{2\pi}/S\right)^{n_{rep}}\left(\sqrt{2\pi}/T\right)}\int_{\mu} \exp\left(-\frac{1}{2}(S^{2}n_{rep}\mu^{2}-2S^{2}\mu\sum_{j} x_{j}+T^{2}\mu^{2}-2T^{2}\mu M)\right)d\mu$$

$$=\frac{\exp(-\frac{1}{2}(S^{2}\sum_{j} x_{j}^{2}+T^{2}M^{2}))}{\left(\sqrt{2\pi}/S\right)^{n_{rep}}\left(\sqrt{2\pi}/T\right)}\int_{\mu} \exp\left(-\frac{1}{2}(S^{2}n_{rep}+T^{2})(\mu^{2}-2\mu\frac{(S^{2}\sum_{j} x_{j}+T^{2}M)}{(S^{2}n_{rep}+T^{2})})\right)d\mu$$

$$\begin{matrix} =\frac{\exp(-\frac{1}{2}(S^{2}\sum_{j} x_{j}^{2}+T^{2}M^{2}))}{\left(\sqrt{2\pi}/S\right)^{n_{rep}}\left(\sqrt{2\pi}/T\right)}\exp(\frac{(S^{2}n_{rep}x_{mean}+T^{2}M)^{2}}{2(S^{2}n_{rep}+T^{2})}) \\ \int_{\mu} \exp\left(-\frac{1}{2}(S^{2}n_{rep}+T^{2})(\mu-\frac{(S^{2}n_{rep}x_{mean}+T^{2}\mu)}{(S^{2}n_{rep}+T^{2})})^{2}\right)d\mu\end{matrix}$$

$=\frac{\exp(-\frac{1}{2}(S^{2}\sum_{j} x_{j}^{2}+T^{2}M^{2}))}{\left(\sqrt{2\pi}/S\right)^{n_{rep}}\left(\sqrt{2\pi}/T\right)}\exp(\frac{(S^{2}n_{rep}x_{mean}+T^{2}M)^{2}}{2(S^{2}n_{rep}+T^{2})})\frac{\sqrt{2\pi}}{\sqrt{S^{2}n_{rep}+T^{2}}}$.

Therefore, the marginal likelihood is:

$P(\mathbf{X}|C)=\frac{\sigma}{\left(\sqrt{2\pi}\sigma\right)^{n_{rep}}\sqrt{n_{rep}\tau^{2}+\sigma^{2}}}\exp-(\frac{\sum_{j} x_{j}^{2}}{2\sigma^{2}}+\frac{M^{2}}{2\tau^{2}})\exp(\frac{(\frac{\tau^{2}n_{rep}^{2}x_{mean}^{2}}{\sigma^{2}}+\frac{\sigma^{2}M^{2}}{\tau^{2}}+2n_{rep}x_{mean}M)}{2(n_{rep}\tau^{2}+\sigma^{2})})$.
