# Supplementary material for: A hierarchical Naïve Bayes Model for handling sample heterogeneity in classification problems: an application to tissue microarrays
Source: BMC Bioinformatics. 2006 Nov 24;7:514. doi: 10.1186/1471-2105-7-514 (PMC1698579; doi:10.1186/1471-2105-7-514)
Supplement: Additional file 2 — Parameter values used to generate simulated data. [file 1471-2105-7-514-S2.doc]

### Additional file 2 – Parameter values used to generate the second set of simulated data

|  |  | Class 1 | | | Class 2 | | |
| --- | --- | --- | --- | --- | --- | --- | --- |
| **Exp** | N Feat. | M | τ^2^ | σ^2^ | M | τ^2^ | σ^2^ |
| **1** | 1 | 90 | 300 | 100 | 140 | 600 | 400 |
| **2** | 2 | 90 | 300 | 100 | 140 | 600 | 400 |
|  |  | 120 | 350 | 100 | 155 | 500 | 400 |
| **3** | 3 | 90 | 300 | 100 | 140 | 600 | 400 |
|  |  | 120 | 350 | 100 | 155 | 500 | 400 |
|  |  | 60 | 300 | 300 | 80 | 100 | 100 |
| **5** | 10 | 90 | 300 | 100 | 140 | 600 | 400 |
|  |  | 120 | 350 | 100 | 155 | 500 | 400 |
|  |  | 60 | 300 | 300 | 80 | 100 | 100 |
|  |  | 105 | 300 | 100 | 120 | 600 | 400 |
|  |  | 120 | 300 | 100 | 155 | 500 | 400 |
|  |  | 90 | 300 | 100 | 120 | 600 | 400 |
|  |  | 150 | 350 | 100 | 170 | 500 | 400 |
|  |  | 65 | 300 | 150 | 85 | 500 | 300 |
|  |  | 105 | 300 | 100 | 120 | 600 | 400 |
|  |  | 120 | 300 | 100 | 130 | 600 | 300 |

TABLE LEGEND:. Exp=number of experiment, N. Feat= Number of features, M=class mean; *τ^2^*=class variance; *σ^2^*=averaged *within* sample variance.

The results corresponding to these experiments are reported in Table 2.
