# Supplementary material for: A hierarchical Naïve Bayes Model for handling sample heterogeneity in classification problems: an application to tissue microarrays
Source: BMC Bioinformatics. 2006 Nov 24;7:514. doi: 10.1186/1471-2105-7-514 (PMC1698579; doi:10.1186/1471-2105-7-514)
Supplement: Additional file 3 — Comparison of the proposed approach with other classification strategies on the TMA protein expression dataset. [file 1471-2105-7-514-S3.doc]

### Additional file 3 – Comparison of the proposed approach with other classification strategies on the TMA protein expression dataset

We have compared the hierarchical Naïve Bayes classifier (HierNB) with 6 well known classification algorithm on the real TMA data presented in the paper. The methods are: Standard Naïve Bayes with gaussian distribution (StNB), Standard Naïve Bayes with discretization and multinomial distributions (StNB-Dis), Support Vector Machines with linear kernel (SVM), Logistic regression, Classification trees, K-nearest neighbour and Majority Voting. We have exploited the classification algorithms implemented in the software Orange Package (University of Ljubliana, Slovenia)(http://www.ailab.si/orange). Results refer to leave one out cross validation. Since not all the algorithms output a probability, the Brier score and Area under ROC curve have been omitted.

|  | **Acc** | **Spec** | **Sens** | **AUC** | **Brier** |
| --- | --- | --- | --- | --- | --- |
| **HierNB** | 0.65 | 0.71 | 0.60 | 0.69 | 0.41 |
| **StNB** | 0.58 | 0.58 | 0.57 | 0.61 | 0.47 |
| **SVM** | 0.61 | 0.63 | 0.59 | 0.60 | 0.78 |
| **Logistic regression** | 0.61 | 0.63 | 0.59 | 0.61 | 0.48 |
| **Classification tree** | 0.54 | 0.60 | 0.47 | 0.51 | 0.75 |
| **K-nearest neighbour** | 0.47 | 0.43 | 0.47 | 0.43 | 0.98 |
| **Majority** | 0.51 | 1 | 0 | NA | 0.51 |

TABLE LEGEND: Acc=Accuracy, Spec=specificity, Sens= Sensitivity, AUC= area under the ROC; Brier=Brier Score; NA=Not Available.

It is possible to note that the proposed HierNB method outperforms all the others on this real dataset. We can also note that the method has the best performance in terms of specificity, which is one of the main goal in this prediction problem (avoiding overtreatment of patients). It also appears that the HierNB method is the most robust, as shown by the highest AUC value.
