# Supplementary material for: A hierarchical Naïve Bayes Model for handling sample heterogeneity in classification problems: an application to tissue microarrays
Source: BMC Bioinformatics. 2006 Nov 24;7:514. doi: 10.1186/1471-2105-7-514 (PMC1698579; doi:10.1186/1471-2105-7-514)
Supplement: Additional file 4 — ROC Curves for the TMA protein expression dataset as calculated by running 100 times 10-fold cross validation (Table 4 in the paper). [file 1471-2105-7-514-S4.doc]

**Additional file 4**

**ROC Curves for the TMA protein expression dataset as calculated by running 100 times 10-fold cross validation (Table 4 in the paper)**

**
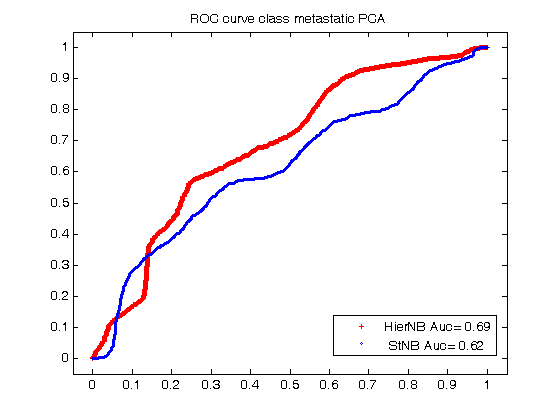
**
